# A Rare Surgical Site Infection Caused by Leifsonia aquatica (Non-diphtheric Corynebacterium) in an Immunocompetent Patient: A Case Report From North India

**DOI:** 10.7759/cureus.70295

**Published:** 2024-09-26

**Authors:** Ankur Goyal, Shweta Singhal, Vikas Kumar, Pragya Shakya, Sapna Goyal

**Affiliations:** 1 Department of Microbiology, Sarojini Naidu Medical College, Agra, IND

**Keywords:** antibiotic susceptibility, immunocompetent patients, leifsonia aquatica, non-diphtheric corynebacterium, surgical site infection

## Abstract

Here we present a case report on a *Leifsonia aquatica* (*L. aquatica, *non-diphtheria *Corynebacterium*) infection in an apparently immunocompetent patient. The organism was isolated from the collection of a lesion in the thigh that presented 15 days post surgery of a fractured femur. The patient had undergone a surgical procedure for a fractured femur with an external device placed, after which she developed pus discharge from the surgical site. This discharge was not associated with any other symptoms such as fever, malaise, chills, or rigor. Pus was processed conventionally for culture, and sensitivity and identification of organisms were done on an automated system. The organism isolated from the sample was identified as *L. aquatica,* which is a non-diphtheria coryneform bacteria that is considered a rare cause of infection. It was susceptible to vancomycin, linezolid, teicoplanin, and quinolones. The present case report describes the rare case of *L. aquatica* from surgical site infection.

## Introduction

Coryneform bacteria are aerobic, irregularly shaped, and non-spore-forming Gram-positive rods. Some coryneform species are toxigenic, like *Corynebacterium diphtheriae*, which may pose a major public threat, and some are non-diphtheria *Corynebacterium*, which usually form part of normal human flora. It is very difficult to speciate non-diphtheria *Corynebacterium *conventionally in clinical labs, and the presence of non-diphtheria *Corynebacterium* is usually considered a contaminant in clinical specimens. Therefore, it has not received a great deal of attention in clinical practice [1]. Recently, due to many advancements in identification, various non-diphtheria *Corynebacterium *are now increasingly recognized as a cause of significant human infection. Many published case reports have shown *Corynebacterium *amycolatum, *Corynebacterium aurimucosum*, *Corynebacterium jeikium*, *Corynebacterium striatum*, *Corynebacterium urealyticum*, and *Leifsonia aquatica* (*L. aquatica*) as causes of human infection in immunocompromised hosts [2, 3]. *Leifsonia aquatica* is a motile, non-spore-forming gram-positive rod. It was first identified by Einar Leifson in 1962 as *Corynbacterium aquaticum* [4]. It is found in environmental water habitats. Infections due to *L. aquatic*a are rarely reported as catheter-related bloodstream infections in immunocompromised patients. Macroscopically, *L. aquatica* grows as opaque and butyrous colonies, which often produce a yellow-green pigment after extended incubation. This organism is motile by peritrichous flagella and is catalase and oxidase positive [5]. We hereby present a rare case of a surgical site infection in an immunocompetent individual due to *L. aquatica* as the cause of infection.

## Case presentation

**Figure 1:**
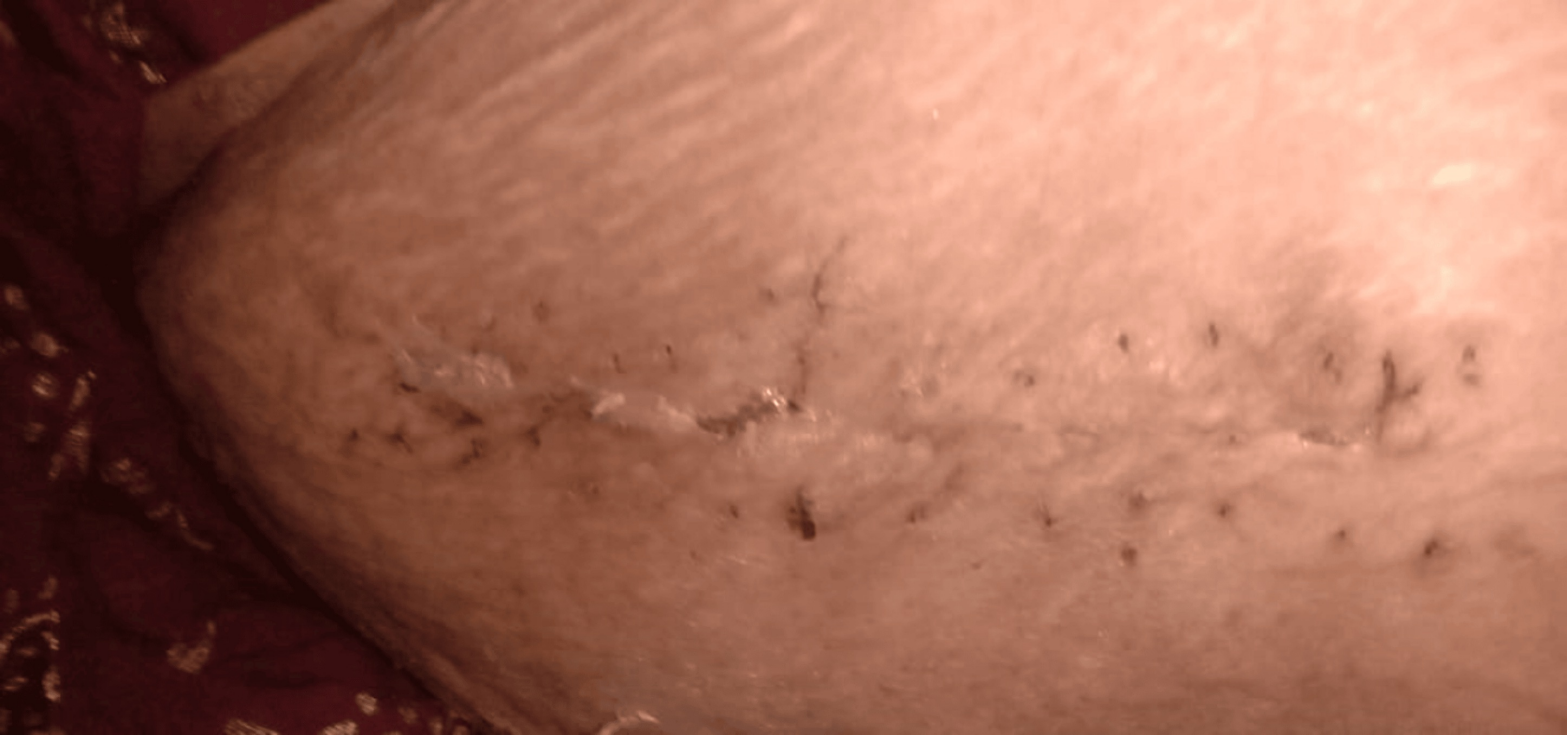
Surgical site from where pus discharge was noted

The patient was a 66-year-old female who fell from a stool 17 days before presentation. She suffered fractures of the humerus and femur. She was non-diabetic, non-hypertensive, and otherwise immunocompetent. She was then advised to undergo open reduction for the fractured femur. On day three of her fall, she underwent a surgical procedure under all aseptic precautions for her fracture, with plating done as shown in Figure 1.

**Figure 2:**
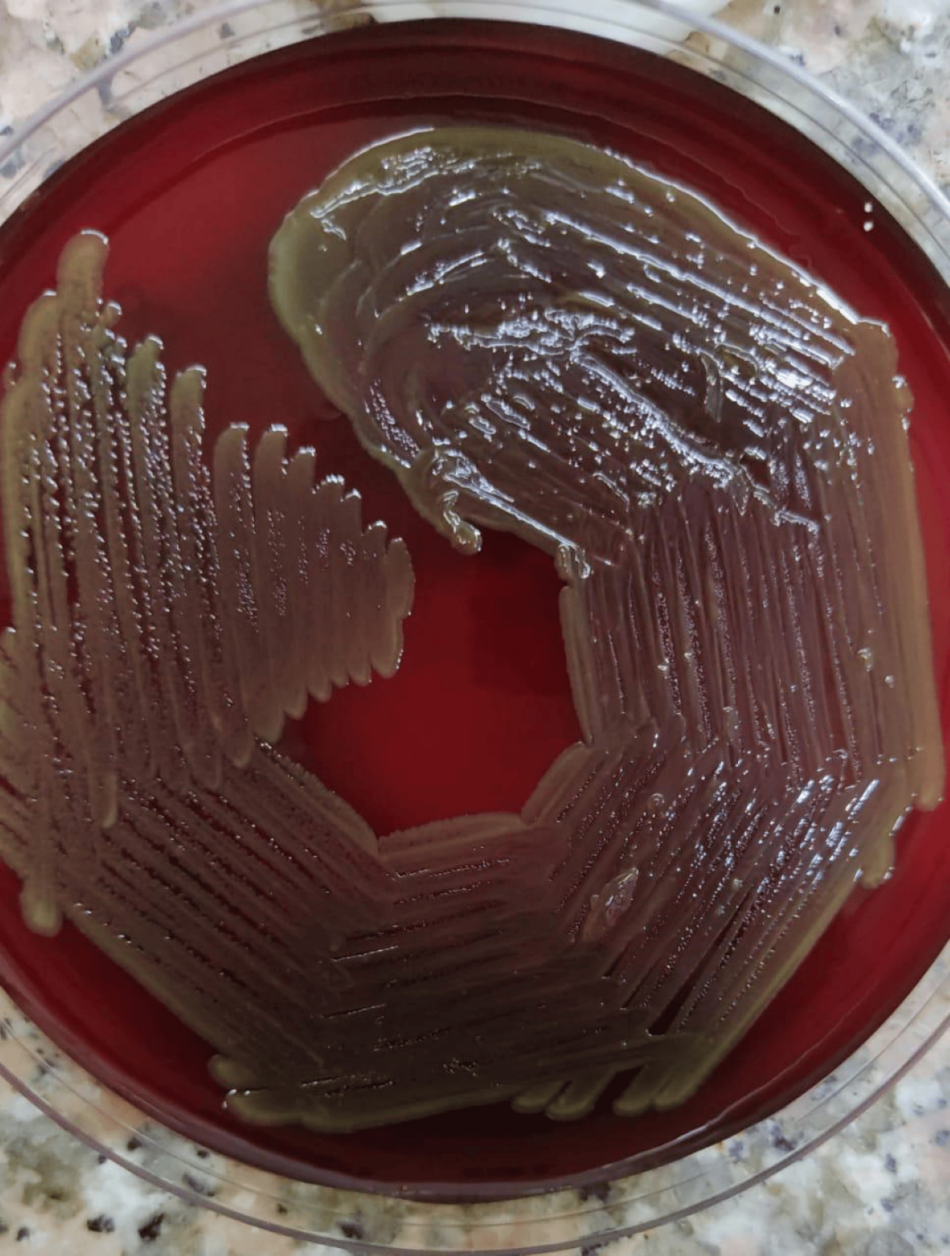
The blood agar plate shows growth of Leifsonia aquatica

The surgery and postoperative period were uneventful, and the patient was discharged on some medication. On day 14 of surgery, during her regular dressing procedure, the patient’s attendant noticed some pus discharge from the surgical site. On examination by the surgical team, there was a frank collection of pus along with pain at the surgical site. Other localized or systemic symptoms such as fever, malaise, etc. were not present. Around 15 ml of fluid (pus) was drained from the surgical site and sent for microbiological examination. Macroscopically, pus was fluid in consistency, reddish in color, and odorless. Immediately after receiving the sample in the lab, it was inoculated on blood agar, MacConkey agar, and brain heart infusion (BHI) broth. On direct microscopic examination, it was negative for acid-fast bacilli, and no fungal elements were seen on the potassium hydroxide (KOH) wet mount. On Gram staining, plenty of pus cells with few small Gram-positive bacilli were found. After overnight incubation, there was no growth on MacConkey agar, but on blood agar, smooth, shiny colonies were grown (Figure 2).

On prolonged incubation of 48 hours, colonies became yellow-green colored. It was catalase- and oxidase-positive. It was giving the appearance of *Pseudomonas *species (smooth, moist, and pigmented), but no growth was observed on MacConkey agar, which suggested that it is not a *Pseudomonas *species. On Gram staining, it was small Gram-positive bacilli. After overnight incubation, the BHI broth was also turbid. It was also subcultured on blood and MacConkey agar. Microscopically turbid BHI broth also showed small gram-positive rods. The next day, on subculture, the same type of single growth was observed in blood agar and no growth on MacConkey agar. The identification of the organism was done using an automated BD Phoenix system (Becton, Dickinson, and Company, Franklin Lakes, NJ), which identified the organism as *L. aquatica.* Since the growth on blood agar was single-type, it was reported as a causative organism for the infection. Antimicrobial susceptibility testing for the organism was performed conventionally by the Kirby-Bauer disk diffusion method as per standard guidelines, and a panel for Gram-positive organisms was set up, and the organism was susceptible to vancomycin, linezolid, teicoplanin, levofloxacin, moxifloxacin, and ofloxacin.

At the time of presentation, the patient had been on cefuroxime-clavulanic acid and clindamycin post surgery. After receiving the culture report, the clinician stopped these antibiotics and started linezolid (600 mg twice a day (BD)) and levofloxacin (500 mg BD). The patient improved clinically after antibiotic change. Discharge became minimal after one week of antibiotic treatment; another swab was taken from the wound site, and culture and sensitivity were performed. No growth was observed in the sample even after incubating in the BHI broth. The patient was still on linezolid and levofloxacin medication. The patient was given three weeks of antibiotic treatment and was observed three weeks after completing the antibiotic treatment. After the full course of treatment, there was no pus discharge. Her hematology and biochemistry investigations (Table 1), suggested that at the time of presentation of pus discharge, her leukocyte count was increased, but after the treatment was given, her leukocyte count improved.

**Table 1:** Hematology and biochemistry investigation Hb: hemoglobin; TLC: total leukocyte count; BUN: blood urea nitrogen

DATE	Hb (g/dl) (reference range: 11-17.3 g/dl)	TLC (/cmm) (reference range: 4,000-11,000/cmm)	Platelets count (lac/cmm) (reference range: 1.5-4.0 lac/cmm)	BUN (mg/dl) (reference range: 7-30 mg/dl)
June 23, 2024	7.2	17,460	3.87	22
June 25, 2024	9.2	14,900	3.34	-
June 27, 2024	7.5	13,130	3.24	34
June 29, 2024	8.1	9,340	3.46	-

## Discussion

Non-diphtheria *Corynebacterium *species are increasingly being reported as the causative agent of clinical infection in the last two decades. Due to the inappropriate use of antibiotics, many times they became multidrug-resistant (MDR) [3]. In the present case report, we found *L. aquatica*, a non-diphtheria *Corynebacterium*, as a causative agent for surgical site infection after a surgical procedure with an implant placed in a femur fracture. We did not find any case reports showing *Leifsonia *as a cause of surgical site infection. There are only a few reports in the literature describing *L. aquatica* as a cause of septicemia in an elderly patient with diabetic ketoacidosis [6], peritoneal dialysis peritonitis [7], neonatal meningitis [8], neonatal urinary tract infection [9], and septicemia in a patient with acute lymphoblastic leukemia [10].

In some reports, like the study done by Dempsey et al., the authors inferred that *Leifsonia* spp. was the most common organism to cause prosthetic joint infection based on DNA sequencing findings. However, they have not been able to isolate *Leifsonia *[11]. In a literature review by Carvalho et al. from 1975 to 2017, there was no report showing that surgical site infection is caused by *Leifsonia *[12]. Identification of these coryneform Gram-positive rods as pathogens in diagnostic laboratories is always difficult. Probably, or to the best of our knowledge, this is the first case report of *L. aquatica* suggesting that an organism may have a significant role in surgical site infection by having an implant as foreign material.

*Leifsonia aquatica* has properties of formation of biofilm and low growth rate; it can pass through polycarbonate water filters, which contribute to the pathogenicity of this bacterium with exogenous devices [11, 13]. Vancomycin is considered an antibiotic of choice [14]. In the present case, the strain was susceptible to vancomycin but resistant to many other antibiotics. Here patient was given linezolid and levofloxacin as per sensitivity report for that particular strain as patient has denied the injectable option. However, due to the propensity to develop biofilm on foreign material, it is highly advisable to use vancomycin to have a complete cure. Some studies have clearly recommended vancomycin as a better option for catheter-related *Corynebacterium *infections. However, in the present case, the patient was completely cured by linezolid and levofloxacin as per the sensitivity report. Therefore, correct identification and their antibiotic sensitivity are key for proper management. 

## Conclusions

We know that postoperative surgical site infections are an important complication of any surgery. Patients should be very vigilant for any discharge from the surgical site. Many times patients are given empirical therapy based on previous experiences, and *Corynebacterium *is never considered a cause of infection as it is considered to be a nonpathogenic organism/contaminant. This report clearly provides evidence that *L. aquatica*, an environmental coryneform bacteria, can cause serious infection in healthy people and should be considered when isolated from clinically relevant specimens. The present case was treated successfully through linezolid and levofloxacin without the removal of the implant. However, the capability of the organism to produce biofilm must be kept in mind; therefore, the drug of choice in such cases is vancomycin. This case study highlights the need for the identification of non-diphtheria *Corynebacterium *as a causative pathogen.
